# Methods to account for movement and flexibility in cryo-EM data processing

**DOI:** 10.1016/j.ymeth.2016.03.011

**Published:** 2016-05-01

**Authors:** S. Rawson, M.G. Iadanza, N.A. Ranson, S.P. Muench

**Affiliations:** a School of Biomedical Sciences, Faculty of Biological Sciences, University of Leeds, Leeds LS2 9JT, UK; b School of Molecular and Cellular Biology, Faculty of Biological Sciences, University of Leeds, Leeds LS2 9JT, UK

**Keywords:** Cryo-EM, RELION, Image processing, Motion correction

## Abstract

Recent advances in direct electron detectors and improved CMOS cameras have been accompanied by the development of a range of software to take advantage of the data they produce. In particular they allow for the correction of two types of motion in cryo electron microscopy samples: motion correction for movements of the sample particles in the ice, and differential masking to account for heterogeneity caused by flexibility within protein complexes. Here we provide several scripts that allow users to move between RELION and standalone motion correction and centring programs. We then compare the computational cost and improvements in data quality with each program. We also describe our masking procedures to account for conformational flexibility. For the different elements of this study we have used three samples; a high symmetry virus, flexible protein complex (∼1 MDa) and a relatively small protein complex (∼550 kDa), to benchmark four widely available motion correction packages. Using these as test cases we demonstrate how motion correction and differential masking, as well as an additional particle re-centring protocol can improve final reconstructions when used within the RELION image-processing package.

## 1 Introduction

Movement of sample molecules during data acquisition can reduce the overall quality of electron micrographs and lower the final achievable resolution of any reconstruction from the data [1]. Movement occurs on multiple levels and to some extent is unavoidable. Mechanical drift of the microscope stage generally results in uniform mono-directional movement of all the particles in each micrograph. Beam induced motion, whether caused by movement of the particles or localized deformation of the ice, affects each particle in the image differently [2]. Moreover, flexibility in the particle, which is independent of beam induced motion or mechanical drift, can allow for different regions of the molecule to move relative to each other. With a single long exposure, the final image is the sum of all these motions, which reduces the quality of the final data.

The ability to record multi-frame ‘movies’ provides new opportunities for processing of electron micrographs after acquisition, allowing the final reconstructions from these data to reach higher resolutions [3]. This has proved especially powerful in correcting for motion at the sample level during data acquisition. When a multi-frame movie is recorded movement vectors for the entire micrograph, as well as individual particles or subdomains, can be calculated and the individual frames, or rolling averages of the frames, aligned computationally, improving the quality of the summed micrograph [2], [4], [5]. Beam induced motion has uneven effects both spatially and temporally. Different regions of the ice may show dramatically different amounts of movement, and larger movements are generally observed in the early frames of a movie. The ability to correct the motions of each individual particle, as well as determine if early frames have large motions and choose to exclude them, can lead to significant improvements in data quality. These techniques have been applied to several recent high-resolution reconstructions including γ-secretase, rotavirus VP6 and TRPA1 [6], [7], [8]. Frame alignment techniques cannot account for movement of subdomains of a particle relative to each other and alternative strategies are required to correct for this. To tackle these kind of motions selective masking techniques can be used which focus on specific domains that are known to be a rigid body relative to other domains. As the smaller domains often have poor signal to noise its important that the particles are aligned on the whole molecule in the first instance with selective masking carried out as a subsequent step.

Several software packages are available to perform both whole frame and individual particle motion correction. Motioncorr [9] and Unblur [7] can perform whole frame motion correction, whilst the reconstruction package RELION [10] has integrated per particle motion correction [11] and the alignparts_LMBFGS (Limited Memory Broyden–Fletcher–Goldfarb–Shanno) algorithm [12] and Unblur are also able to perform correction of a per particle basis. Although all of these software packages are designed to perform similar functions, transfer of data between the different programs and integrating them into the RELION workflow can sometimes be difficult, especially in modification of the required “star” file.

Here we attempt to benchmark several techniques for motion correction and demonstrate how they can be integrated into the RELION workflow to improve the quality of reconstructions from a diverse set of samples. Several scripts written in Python are provided to allow for the transfer of data back and forth between RELION and outside programs. Additionally a script for particle re-centring, as described by Ge et al. [13], in RELION is provided which, although unrelated to motion correction, improves the centring of the original particles and facilitates downstream processing.

## 2 Methods and results

The following sections will detail each of our pipelines for particle recentring, motion correction and assessing inherent flexibility.

### 2.1 Test datasets

Three sample datasets were chosen for testing (Table 1). All three were collected on a 300 kV Titain Krios (FEI corporation) with a Falcon II direct detector. All micrographs were collected with a total dosage of ∼50 e^−^/Å^2^ over all frames.

### 2.2 Recentring

All single particle reconstructions in cryo-EM begin with picking particles. Individual particles must be identified and windowed out at the centre of a box with a specific size that both encompasses the full particle and provides sufficient background for noise estimation and/or CTF correction depending on the downstream processing package. This is not trivial; the low signal to noise of the images makes manual picking difficult and tedious, especially for large numbers of particles/micrographs. Many programs such as RELION and EMAN2 have utilities for automated particle picking which must be trained using low resolution templates [14], [15]. No matter the method used, particles must be accurately centred in their boxes, which can be difficult to achieve with either manual or automatic picking.

To address this Ge et al., demonstrated a method to improve the centring of particles by using the translations applied to the particles during alignment to shift the coordinates of the original box used to window out the particle [13]. By processing the newly recentred particles they were able to improve the resolution of their structure from 10 to 6 Å as the increased accuracy of the particle centring allowed the use of a tight soft mask with the recentring only providing marginal resolution improvements on its own.

To recentre particles we first carry out a 3D reconstruction using 3D auto-refine within RELION, which calculates values for the translational shifts in x and y [10]. We then use our own python script, recenter.py, to apply the calculated shifts from the refinement to the particle coordinate file. This allows particles to be re-extracted with improved centring. This recentred stack can then be used for subsequent steps of classification and/or refinement. An added benefit of this procedure is it can also allow the application of a very tight mask to the particles in subsequent processing steps. As the particles are already well centred the probability of the mask cutting into protein density is reduced. This may be particularly advantageous for densely packed micrographs. The script recenter.py (Table 2) is provided to facilitate using this procedure. The script accepts a RELION ‘_data’ star file output from the 3D auto-refine step, which contains data columns “_rlnCoordinateX”, “_rlnCoordinateY”, “_rlnOriginX”, “_rlnOriginY” as input. It outputs a star file with updated x and y coordinates for each micrograph which can then be used in the particle extraction step.

When this re-centring was applied to V_1_ (29,905 particles of a ∼550 kDa soluble protein) the resolution of the resulting model from refinement following post processing increased from 8.7 Å to 8.0 Å. This procedure was then trialled on 24,109 particles of a large ∼4 MDa Qβ virus dataset and resulted in 0.24 Å improvement in resolution before post processing. This suggests the accuracy of the initial centring is more robust for the larger particles. This should generally be true for objects, such as viruses, with a well-defined centre and more even distribution of mass around that centre. Subsequently this procedure probably has less benefit for larger specimens, but may be of greater use for proteins where the shape or distribution of density makes accurate centring more challenging.

### 2.3 Motion correction

As stated previously the ability to capture multiple frames per second that can be merged into a final exposure, is a major advantage of new technologies for recording cryoEM data. This allows for flexibility in post-capture image processing. Choosing to include or exclude specific frames can control the effective dose, and the temporal resolution of these ‘movies’ allows for corrections to be made for several sources of movement within the sample. Motion correction on a whole frame basis can correct for drift or other motions of the stage, moreover motion correction on a per-particle basis allows for correction of local movement, such as beam-induced motion. Some of these techniques are accessible as part of defined packages such as RELION, others are designed as stand alone packages including Unblur [7], Motioncorr [9] and alignframes_lmbfgs and alignparts_lmbgfs [12]. Here we compare these different packages for both whole-frame and per-particle correction in terms of computational time and effect on the final reconstruction. We also provide scripts that can facilitate the use of per particle motion correction with alignparts_lmbfgs and both whole frame per particle correction using Unblur within the RELION workflow. A particle recentering script is also provided which, although not directly related to motion correction, can sometimes improve the per particle results.

#### 2.3.1 Whole frame motion correction

986 movies from V_1_ were processed using Motioncorr, or Unblur both with and without exposure filtering (Table 3). Following this, the 29,905 recentred particles were extracted from the corrected image sums and 3D models produced using the RELION 1.4 3D auto-refinement procedure and post processed. No substantial differences were found in quality of 3D model from each program with differences in wallclock time likely explained by the GPU acceleration of Motioncorr.

Two scripts are provided to automate and streamline this process. Motioncorr.py (Table 4) and unblur-auto.py (Table 5) run Motioncorr and Unblur, respectively, on batches of images and output corrected frames and merged images with RELION compatible nomenclature.

#### 2.3.2 Per-particle motion correction

Per-particle motion correction was carried out using three separate techniques: the particle polishing procedure within RELION, Unblur and alignparts_lmbfgs. Unblur was unable to align the frames on a per particle basis using V_1_ because the small box size (128 × 128) with the relatively small particle did not contain sufficient signal to carry out the alignment. Therefore V-ATPase which is a larger (∼1 MDa) protein complex in a larger box (320 × 320) was used as a test set. It should be noted that this sample was collected on a carbon support film which also diminished the signal to noise ratio.

All micrographs were motion-corrected with Motioncorr before 2D classification with RELION to leave a clean dataset where per-particle motion correction was then performed using each of the three methods (Table 6). Both Unblur and alignparts_lmbfgs were used with the exposure filtering option enabled and B-factor based damage weighting was carried out in RELION’s particle polishing. Due to the low signal to noise within the particles a 5 frame running average was used in the B factor correction damage-weighting step in RELION’s particle polishing. Unblur and alignparts_lmbfgs were run using either default or recommended parameters. Following per particle motion correction within each program, a 3D reconstruction was made using the auto-refinement procedure within RELION and the resulting models compared following postprocessing with the same mask. In the case of alignparts_lmbfgs when the output particle stack was manually inspected several particles appeared as noise images. This is potentially owing to the very low signal in some of the micrographs because of the carbon layer on the grid – indeed the particles from lower defocus micrographs, with lower contrast seemed to be misaligned more often. Previous work [12], [16] has shown this program to be extremely effective demonstrating that different programs may be more or less suitable for an individual dataset. In all cases total wallclock times for carrying out per-particle motion correction are extremely similar.

Two scripts are provided to expedite the process of using Unblur and alignparts_lmbgfs for per-particle motion correction in the RELION workflow. Reorder4LMBFGS.py accepts a RELION star file containing particle coordinates for all of the particles. The script sorts the individual frames and outputs a star file formatted for alignparts_lmbgfs (Table 7). PP-unblur.py accepts a star file containing all of the particles in that particular micrograph for each movie. The script then uses RELION to make a new image stack formatted for Unblur. It then runs Unblur on each image stack and finally writes a single star file containing all of the Unblur corrected particles (Table 8).

### 2.4 Sample flexibility

Accounting for flexibility within a complex is a powerful approach to understand its mechanical properties and can be achieved in both 2D classification and 3D refinement. Previously 2D classification of dynein and the SptP3 for example have revealed conformational flexibility based on 2D classes [17], [18], [19]. Accounting for flexibility during 3D reconstruction can greatly improve the resolution of individual domains as shown for γ-secretase [6], [20]. For all of these studies, inherent flexibility has been overcome through the use of masking out specific regions of interest. For this study we have used the V-ATPase as a model system as it works through a rotary mechanism which involves structural flexibility as seen in 2-D image classes from electron microscopy (EM) [21], and molecular dynamics simulations [22], [23]. Compared to a series of discrete states, the continuous nature of this flexing makes sorting of the heterogeneity difficult by conventional 3-D classification. As an alternative, we investigated whether masking based on V_1_ and V_o_ would improve the resolution of the resulting models.

Micrographs were first motion corrected with Motioncorr [9] before particle picking and data processing. A total of 30,730 particles were picked which, after 2-D classification in RELION [10], resulted in 13,083 particles in well-defined classes. The 3-D reconstruction had a tighter mask than the one which we used to generate our ∼1 nm published reconstruction, which resulted in a similar global resolution of 9.2 Å, but with a clear difference in detail between V_1_ and V_o_, as described in [24].

**Fig. 1:**
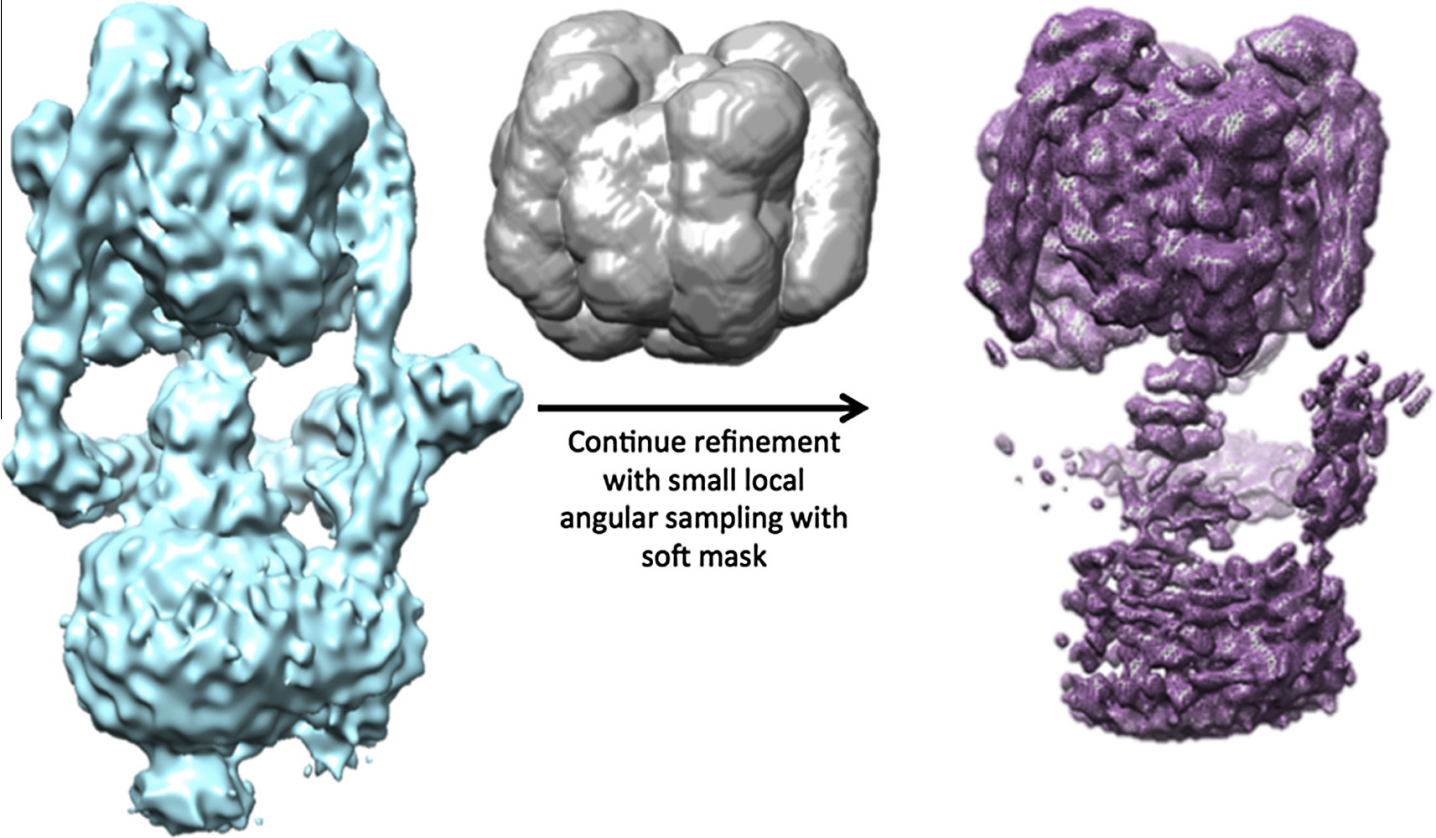
Effect of applying tight soft mask (grey) around the V_1_ domain with local searches starting from previous 3D refinement (blue) to improve resolution and detail in this region (purple).

**Fig. 2:**
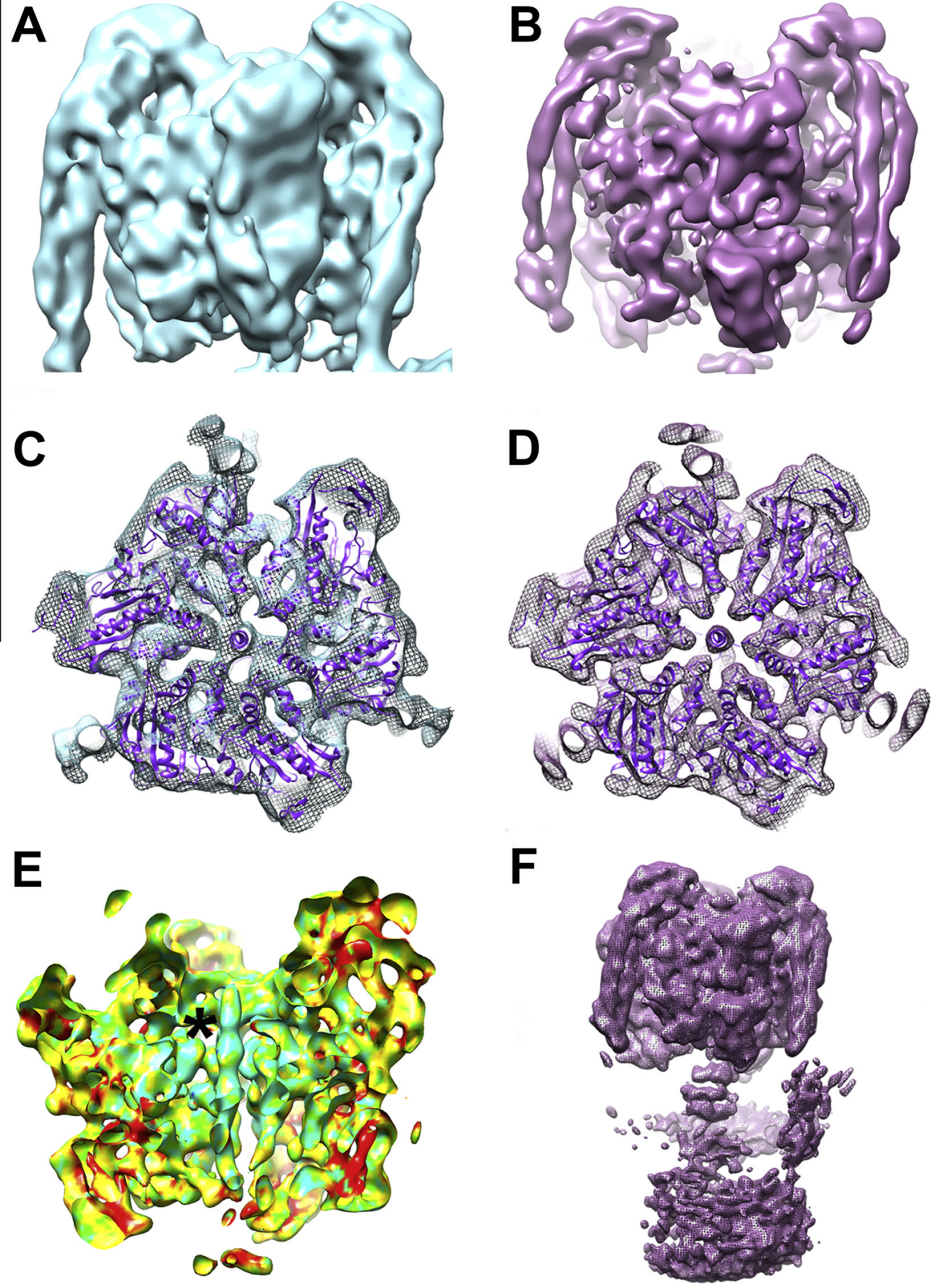
Surface representations and sections through V_1_ calculated from a full V-ATPase mask (A, C) and a mask with only V_1_ (B, D). (E) Local resolution map in the V_1_ masked map; coloured cyan, light green, dark green, yellow and red for 5 Å, 7 Å, 9 Å and 12 Å, respectively (asterisk marks the highest resolution ‘bearing’ region). (F) Full V-ATPase reconstruction calculated from the V_1_ masked data showing how V_o_ becomes more poorly resolved, suggesting the domains are not rigidly linked.

3-D refinement was then carried out separately with the same data on V_1_ and V_o_ through performing local angular searches based around previously determined Euler angles and imposing domain-specific masks in RELION (Fig. 1). Masking, to account for this flexibility resulted in a significant increase in the global resolution of the V_1_ reconstruction (8.2 Å), with a more modest improvement, if any, to V_o_ (∼1 nm). Analysis of the masked V_1_ shows a clear improvement in the resulting reconstruction that is consistent with the higher resolution of 8.2 Å (Fig. 2A-D), with local resolution determination using ResMap showing a range from 5 Å to 12 Å (Fig. 2E), with a significant proportion <7 Å [25]. Highest resolution was about the “electrostatic collar of density” which is the region holding the central rotor axle in place and is proposed to account for the extraordinarily high-energy conversion efficiency of the V-ATPase [24].

The stators show a clear separation of the two α-helices in the masked V_1_, in contrast to the reconstruction obtained masking the whole complex (Fig. 2A, B). However, the base of the stators becomes poorly resolved, as does the central rotor axle and V_o_ domain when the alignment and reconstruction is based on the V_1_ domain, which indicates flexing between the two domains (Fig. 2F). Masking of the central region alone resulted in a low quality reconstruction, likely due to its small volume.

Similar masking of V_o_ gave no significant improvement in the final model with a calculated resolution of ∼1 nm. This could be limited by two factors: firstly heterogeneity within V_o_ or alternatively, the smaller size of V_o_ and the presence of bound detergent makes alignment more challenging. This example shows the limitations of the masking approach – if the area you examine is too heterogeneous or lacking in features to allow accurate alignment then you may not gain any additional information and would perhaps be better served improving the sample preparation, add ligands/proteins which are though to stabilize the region or study this region separately.

## 3 Conclusion

The effectiveness of the techniques described in this paper will vary depending on the data to which they are applied. In some cases we found improvements in resolution of the final maps generated from data treated with some or all of these techniques. The scripts provided allow for interchange between RELION and stand alone motion correction software, which will allow users to test various methods of recentring and of motion correction to find that which best suits the size, shape, and signal to noise of their data. All of the scripts described in the paper are available at https://github.com/leedsEM/movement.

## Figures and Tables

**Table 1:** The three data samples used for this study and the corresponding collected data.

Sample	MW (MDa)	Micrographs	Frames	Particles	Pixel size (Å)
Qβ virus	∼4	1206	32	24,109	1.35
V-ATPase	∼1	1366	34	13,083	1.35
V_1_	∼0.55	986	32	29,905	1.76

**Table 2:** Inputs and outputs for recenter.py.

Input(s)			Output(s)	
File	Source	Description	File	Description
Star file	2D classification or 3D Autorefine step of Relion 1.4/Relion 1.3	Lists particle locations for each frame ordered by frame	Recentered star file	Lists particle locations by frame with x and y shifts applied

**Table 3:** Whole frame motion correction of V_1_ with corresponding processing time, final model resolution and machine spec.

Process	Wallclock time	Final resolution (Å)	Machine spec
Motioncorr	550 min	8.05	GPU: NVIDIA QUADRO K2000 2 Gb, 384 CUDA cores
Unblur	843 min	8.05	Intel Xeon CPU E7 – 4807@1.87 GHz, 24 core, 32 Gb RAM
Unblur + exposure filter	1310 min	8.34	Intel Xeon CPU E7 – 4807@1.87 GHz, 24 core, 32 Gb RAM

**Table 4:** Inputs and outputs for motioncorr.py.

Input(s)			Output(s)	
File	Source	Description	File	Description
Motioncorr	Download^a^	Motion correction software by Li et al. [9]	N/A	N/A
Original movie files	Direct detector	Multiple frame movies 1 file per micrograph	Corrected movie files	Multi-frame movie files with motion correction applied, 1 per micrograph
Corrected sums	Sum of motion corrected frames, 1 per micrograph
Uncorrected sums	Sum of uncorrected frames, 1 per micrograph. Not produced if script is run with –s flag

a Currently available at http://www.nature.com/nmeth/journal/v10/n6/full/nmeth.2472.html.

**Table 5:** Inputs and outputs for unblur-auto.py.

Input(s)			Output(s)	
File	Source	Description	File	Description
Unblur 1.0	Download^a^	Motion correction software from Grant et al. [7]		
Original movie files	Direct detector	Multiple frame movies 1 file per micrograph	Unblurred stacks	One merged mrc file per micrograph

a Currently available at http://grigoriefflab.janelia.org/unblur.

**Table 6:** Per particle motion correction using Relion 1.4 Polish, LMBFGS and Unblur with exposure filtering. The processing time along with machine specifications and final resolution are shown.

Process	Wallclock time	Final resolution/Å (before postprocessing)	Machine spec
Uncorrected	0 min	10.8 (18.0)	N/A
Relion 1.4 Polish	1254 min	9.8 (17.3)	Intel Xeon CPU E5-2670 @ 2.60 GHz, 16 core, 64 Gb RAM
LMBFGS	1202 min	18 (18.8)	Intel Xeon CPU E5-2670 @ 2.60 GHz, 16 core, 64 Gb RAM
Unblur + exposure filter	155 min^a^ 1206^b^	9.8 (18.0)	Intel Xeon CPU E5-2670 @ 2.60 GHz, 16 core, 64 Gb RAM

a Time to run unblur only.

b Time including all processes in PP-unblur.py.

**Table 7:** Inputs and outputs for reorder4LMBFGS.py.

Input(s)			Output(s)	
File	Source	Description	File	Description
Star file	Particle extraction step of Relion 1.4/Relion 1.3	Lists particle locations for each frame ordered by frame	Reordered star files	One star file for each movie file, listing particle locations by frame and formatted for LMBFGS

**Table 8:** Inputs and outputs for PP-unblur.py.

Input(s)			Output(s)	
File	Source	Description	File	Description
Unblur 1.0	Download^a^	Motion correction software from Grant et al. [7]		
Original movie files	Direct detector	Multiple frame movies 1 file per micrograph	Unblurred stacks	One mrc stack file per micrograph
Star file	Particle extraction step of Relion 1.4/Relion 1.3	Lists particle locations for each frame ordered by frame	Reordered star file	Lists particle locations for each frame ordered by particle number

a Currently available at http://grigoriefflab.janelia.org/unblur.
