# Corrigendum to ‘Methods to account for movement and flexibility in cryo-EM data processing’ [Methods 100 (2016) 35–41]

**DOI:** 10.1016/j.ymeth.2016.05.006

**Published:** 2016-07-15

**Authors:** S. Rawson, M.G. Iadanza, N.A. Ranson, S.P. Muench

**Affiliations:** a School of Biomedical Sciences, Faculty of Biological Sciences, University of Leeds, Leeds LS2 9JT, UK; b School of Molecular and Cellular Biology, Faculty of Biological Sciences, University of Leeds, Leeds LS2 9JT, UK

The authors regret that after communication with J. Rubinstein we have re-run alignparts_lmbfgs using a higher lambda value (1d12) than that in the example given in the protocol to take account of the larger image pixel values with a FEI Falcon camera compared to a Gatan K2 summit camera. This has resulted in a significantly improved performance of the program.

The authors would like to apologise for any inconvenience caused.

